# High-resolution mapping of quantitative trait loci controlling main floral stalk length in Chinese cabbage (*Brassica rapa* L. ssp*. pekinensis*)

**DOI:** 10.1186/s12864-019-5810-2

**Published:** 2019-05-30

**Authors:** Shuantao Liu, Ronghua Wang, Zhigang Zhang, Qiaoyun Li, Lihua Wang, Yongqiang Wang, Zhizhong Zhao

**Affiliations:** Institute of Vegetables and Flowers, Shandong Academy of Agricultural Sciences,Shandong Branch of National Vegetable Improvement Center, Shandong Key Laboratory of Greenhouse Vegetable Biology, Vegetable Science Observation and Experiment Station in Huang-Huai Area of Ministry of Agriculture, Ji’nan, 250100 Shandong province People’s Republic of China

**Keywords:** Chinese cabbage (*Brassica rapa* L. ssp. *pekinensis*), High-density map, QTL, Main flower stalk length, Bolting-related trait

## Abstract

**Background:**

For spring-type Chinese cabbage production, premature bolting refers to the excessive elongation of dwarf stems before harvesting. Although quantitative trait loci (QTL) mapping for bolting-related traits have been studied extensively, the main flower stalk length (MFSL) have been rarely investigated. Two inbred lines, 06–247 and He102, have significant differences in the MFSL. In this study, these two materials were selected as parental lines for the construction of a recombinant inbred line (RIL) mapping population. High-density mapping of QTL for the MFSL was performed based on the deep resequencing of parental lines and specific locus-amplified fragment sequencing (SLAF-Seq) of individual recombination inbred lines.

**Results:**

An F_7_ population consisting of 150 lines was developed. Deep resequencing of parental lines produced 21.08 gigabases, whereas SLAF-Seq produced an average of 428.35 million bases for each progeny. The total aligned data from the parental lines identified 1,082,885 high-quality single nucleotide polymorphisms (SNPs) between parental lines. Out of these, 5392 SNP markers with a segregation type of aa×bb and average integrity of > 99% were suitable for the genetic linkage map construction. The final map contained 10 linkage groups (LGs) was 1687.82 cM in length with an average distance of 0.32 cM between adjacent markers. Based on the high-density map, nine QTLs for MFSL were found to be distributed on seven chromosomes, and two major-effect QTLs were identified for the first time. The physical distance between adjacent markers of two major-effect QTLs was 44.37 kbp and 121.91 kbp, respectively. Approximately 2056 and 6769 SNP markers within confidence intervals were identified according to the results of parental line resequencing, which involved 24 and 199 mutant genes.

**Conclusions:**

The linkage map constructed in this study has the highest density in Chinese cabbage to date. Two major-effect QTLs for MFSL in Chinese cabbage were also identified. Among these, a novel QTL associated with bolting mapped on LG A04 was identified based on MFSL. The results of this study provide an important platform for gene/QTL mapping and marker-assisted selection (MAS) breeding for bolting-resistant Chinese cabbage.

**Electronic supplementary material:**

The online version of this article (10.1186/s12864-019-5810-2) contains supplementary material, which is available to authorized users.

## Background

Heading-type Chinese cabbage (*Brassica rapa* L. ssp. *pekinensis*, 2n = 2x = 20), which belongs to the Cruciferae family, is a biennial plant [1]. In China, the area planted with Chinese cabbage accounts for about 15% of the total vegetable cultivated area, ranking first in vegetable cultivated area and yield, respectively [2]. The cultivation system and varieties of Chinese cabbage have been so well developed that year-round cultivation is possible in China. Therefore, the varieties can be divided into three main types according to the culture season, including autumn-type, summer-type and spring-type. However, for spring-type Chinese cabbage cultivation, premature bolting is a persistent problem [3, 4], which often makes leaf-head lack commercial characteristics and results in huge economic losses to farmers. In this study, high-resolution mapping of quantitative trait loci (QTL) controlling the main floral stalk length (MFSL) in Chinese cabbage would provide a valuable basis for effective strategies to obtain bolting-resistant varieties.

With the development in genomics, marker-assisted selection (MAS) has been used widely in crop breeding for enhancing the efficiency of breeding programs [5]. Using this approach, target traits can be selected indirectly using molecular markers that are closely linked to candidate genes or developed from the gene sequences [6]. However, bolting is a complicated trait and regulated by multiple genes that are largely influenced by environmental cues, such as photoperiod and temperature [7]. Thereby it is difficult to identify the genes or tightly linked marker loci required for MAS breeding. In the advent of molecular marker and QTL analyses, a large number of bolting-related QTLs have been identified in *B. rapa* [8–17]. These identified QTLs were based on different segregating populations, in addition, bolting-related traits were evaluated under different environments and locations. Furthermore, the criterion of bolting-related indices is relatively complex. Among the above-stated bolting-related QTL analyses, most of the indices are related to the flowering character such as bolting time (BT, which is the number of days between sowing and bolting, and bolting is judged by visible flower buds that have emerged in 50% of plants in each line) [11, 18], flowering time (FT, which is the number of days from sowing to the first flower opening on each plant) and days-to-flowering (DTF, which is calculated as the day when 50% of the healthy plants had flowered) [14, 15, 17, 19, 20]. A few studies used bolting index (BI) combined with different bolting-related factors such as bud emergence and the length of flower stalk as bolting indicators [13, 17, 21]. Moreover, other investigations used days to bolting (DB) to map the bolting-related QTL, defined as the number of days from seed sowing to 5-cm stalk [16, 17]. To date, no uniform international standard for bolting-related indices has been established.

For spring-type Chinese cabbage production, bolting-related characteristics refer to the elongation of dwarf stem rather than the presence of flower buds. Spring-type Chinese cabbage is mature and ready for sale. If the shorter dwarf stem emerged with buds, their commodity value will not be affected, but if longer dwarf stem emerged without buds, their commodity value will not be lost. Thus, the length of dwarf stem is the most important characteristic with regard to bolting-related traits. During reproductive stage, the dwarf stem develops into the main flower stalk. The elongation of dwarf stem to the main flower stalk is the process of bolting, which is greatly influenced by the environment factors such as temperature and photoperiod. In the present study, a *B. rapa* high-density linkage map were constructed by using specific-locus amplified fragment sequencing (SLAF-Seq) approach of a recombinant inbred line (RIL) population. Since SLAF-Seq is a recently developed low-cost and high-efficient method of high-throughput sequence-based technique, which greatly reduces the complexity of high-quality reference genome libraries [22]. This technology exhibits significant advantages in high-throughput SNP (single nucleotide polymorphism) marker discovery and genotyping for constructing genetic map. In addition, several QTLs for MFSL under two growth conditions were identified. This is the first report for QTLs mapping of MFSL in *B. rapa*.

## Results

### Phenotypic analysis of MFSL in a Chinese cabbage RIL population

**Fig. 1:**
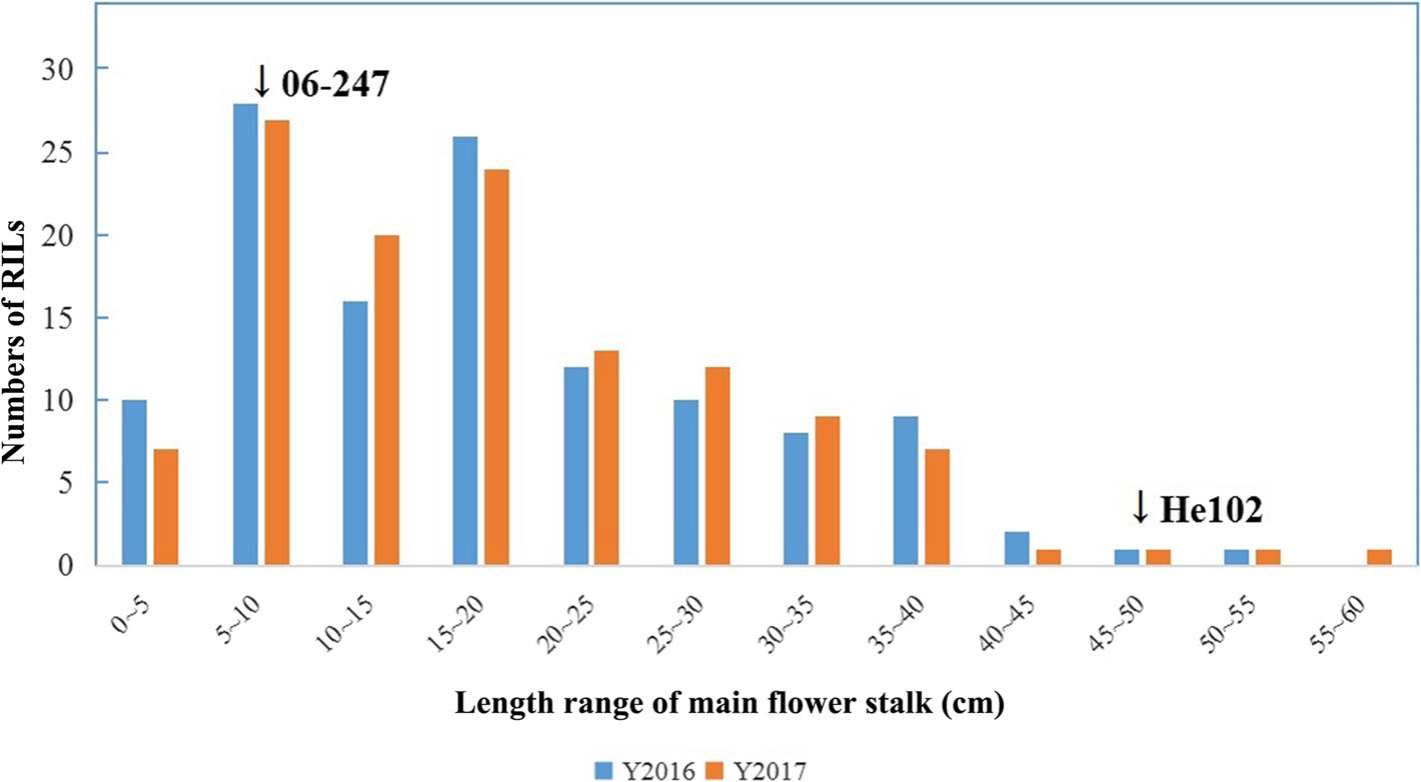
The frequency distribution of MFSL in an RIL population during 2016 and 2017

The phenotypic scores of the two parents and the RIL population were measured (Additional file 1: Table S1). The phenotypic performance of the RIL population was continuously distributed and relatively consistent during the two years, suggesting that the trait was inherited in a quantitative manner. The phenotypic data of the RIL population was highly variable, ranging from 3.00 cm to 55.50 cm and 4.10 cm to 66.30 cm in 2016 and 2017, respectively. While MFSL between year 2016 and year 2017 was highly correlated (r = 0.90). The results of frequency distribution analysis of MFSL are shown in Fig. 1. A comparison of the parental lines showed significant differences (*p* < 0.01) within two years of cultivation (Fig. 1).

### High-throughput sequencing and genotyping of the RIL population

For the two parental lines, He102 and 06–247, the average sequencing read number was 70.40 million, representing 21.08 gigabases (Gb). The Q30 ratio was 90.65%, and average GC content was 38.30%. The average mapped reads and genome coverage for the two parental lines was 97.12 and 86.63%, respectively. However, for each progeny, the average read number was 2.14 million, representing 428.35 million bases. The Q30 ratio was 95.06%, and average GC content was 40.98% for the RI line (Table 1). The total number of aligned reads from the parental lines identified a total of 1,082,885 high-quality SNPs between parental lines, which included 805,813 SNPs that matched the aa×bb segregation pattern. After filtering out lower-quality SNP markers, a total of 5392 high-quality SNP markers were used for linkage map construction. The average sequencing depth for the markers was 33.73× in the parental lines and 27.98× in the offspring, and the average integrity was 99.96%.

**Table 1:** Statistical analysis of sequencing reads from for all samples in Chinese cabbage

Sample	Total number of reads	Total number of bases	Q30 (%)	GC (%)	Mapped (%)	Coverage (%)
He102	72,187,239	21,612,799,598	90.56	38.16	96.77	87.53
06–247	68,608,266	20,544,790,130	90.73	38.44	97.46	85.73
Offspring on average	2,142,942	428,350,920	95.06	40.98	95.42	40.14

Offspring on average: All data were at the average level for offspring

Q30: Sequencing quality score = 30, indicating a 0.1% chance of an error, and thus 99.9% confidence

GC (%): Bumber of G and C/Total base × 100%

Mapped (%): The number of reads that could be aligned onto *Brassica rapa* genome V1.5/Total number of reads from the corresponding line × 100%

Coverage (%): Region covered by mapped reads/Whole reference genome × 100%

### Construction of a high-density linkage map

**Fig. 2:**
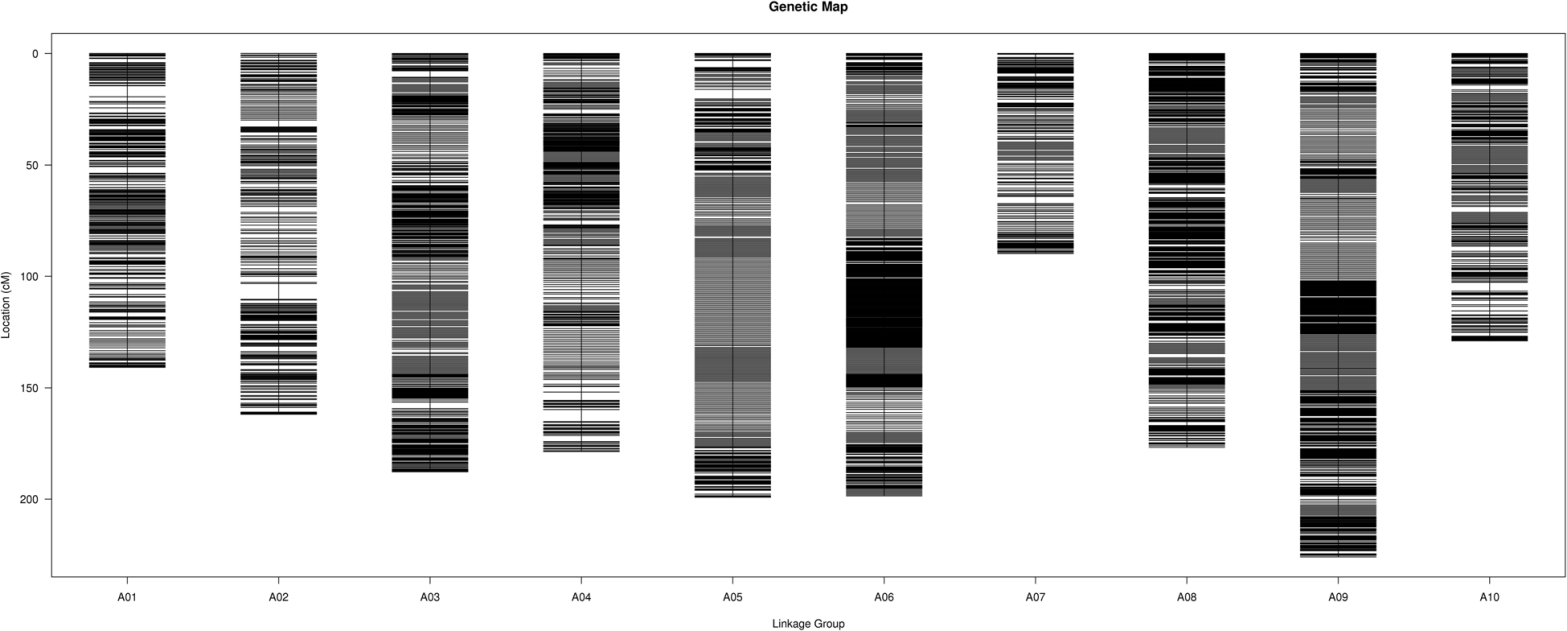
High-density genetic map of MFSL in a Chinese cabbage RIL population. The map includes 10 linkage groups. The name of the linkage group corresponds to that of chromosome. The black bars on the linkage groups represent SNP markers. The crudeness black bar means several SNP markers spaced closely

A total of 5392 markers were assigned to 10 linkage groups (LGs) (Fig. 2). The map spanned a total of 1687.82 cM, with an average distance of 0.32 cM or 49 kbp between adjacent markers (Table 2). The largest LG was LG A09 with 811 markers and a total length of 225.79 cM and an average distance of 0.28 cM between adjacent markers. The smallest LG was LG A07 with 294 markers and a length of 89.76 cM. On the other hand, the marker density could be deduced based on physical distance. For that matter, the densest LG was LG A08, with average physical distance of 36.62 kbp between adjacent markers, while the least dense LG was LG A07, with average physical distance of 88.01 kbp between adjacent markers. The degree of linkage between markers was reflected by a gap ≤5 cM ranging from 99.81 to 100%, with an average value of 99.96%. The largest gap on the map was 7.06 cM, located within LG A02.

**Table 2:** Summary of 10 linkage groups of a high-density genetic map

Linkage group ID	Total number of markers (SDM included)	Total distance (cM)	Average distance (cM)	Max gap (cM)	Gap < 5 cM (%)
A01	490 (6)	140.78	0.29	4.94	100.00
A02	526 (19)	161.87	0.31	7.06	99.81
A03	656 (13)	187.75	0.29	2.89	100.00
A04	490 (6)	178.61	0.37	5.37	99.80
A05	518 (31)	199.12	0.39	4.25	100.00
A06	678 (0)	198.56	0.29	1.48	100.00
A07	294 (16)	89.76	0.31	3.22	100.00
A08	569 (0)	176.72	0.31	1.93	100.00
A09	811 (3)	225.79	0.28	1.55	100.00
A10	360 (5)	128.86	0.36	3.97	100.00
Total	5392 (99)	1687.82	0.32	7.06	99.96

Total number of markers (SDMs included): Total number of markers within the linkage group, the number in brackets indicates the number of segregation distortion markers in the LG

Analysis of the segregation of all 5392 mapped loci showed that 5239 (98.16%) significantly deviated (*P* ≤ 0.05) from the expected 1:1 Mendelian segregation ratio. Only 99 markers showed significant (0.05 < *P* < 0.001) segregation distortion. These segregation distortion markers (SDMs) were located unevenly within the 10 LGs (Table 2). LG A05 had the highest number of SDMs at 31, and LG A08 had no SDMs.

**Fig. 3:**
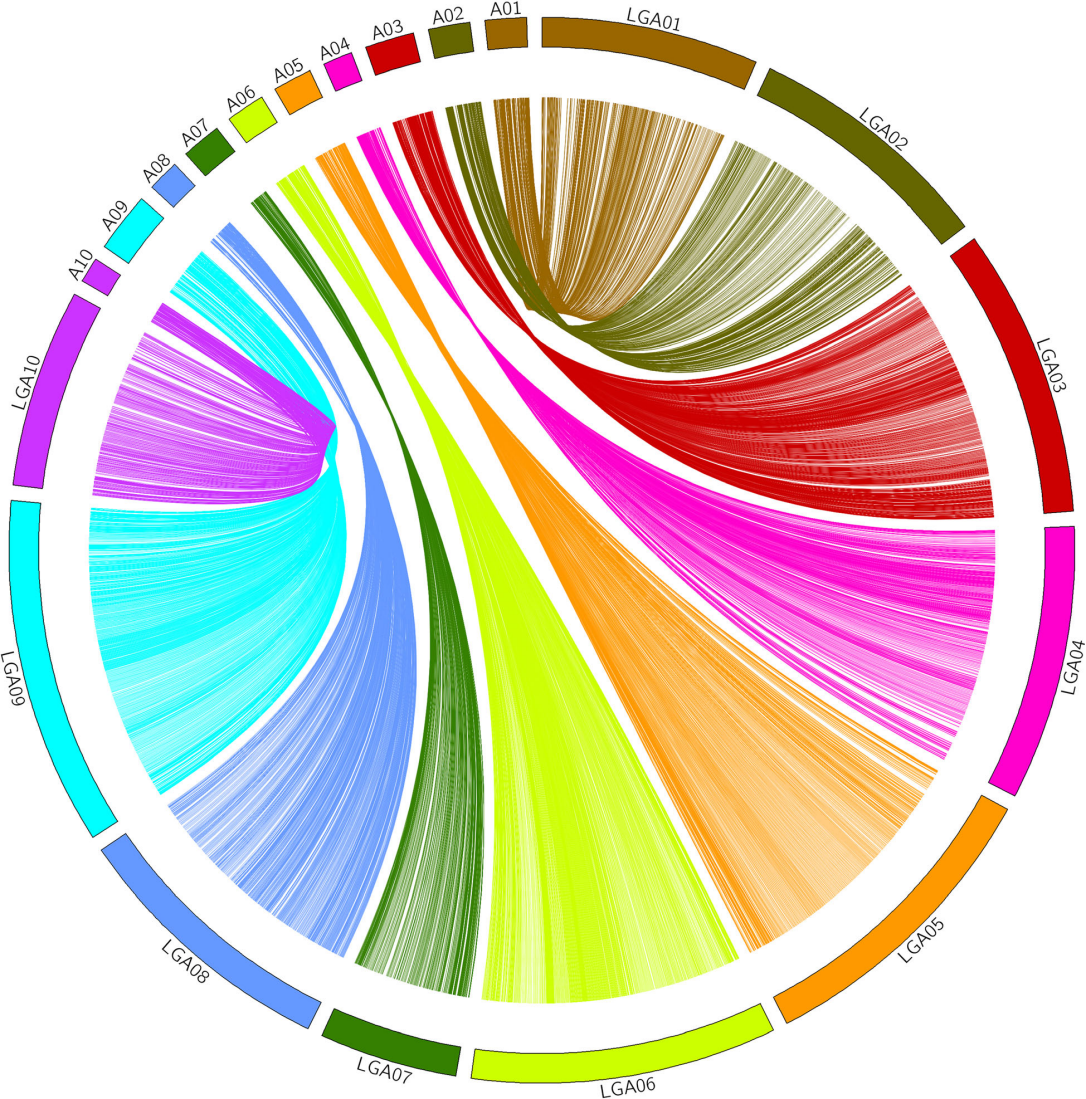
Collinearity comparison between the genetic and physical maps. A01-A10 correspond to physical maps, while LG A01-LG A10 correspond to linkage maps, respectively

Correlation analysis between the genetic location and the corresponding physical position of mapped SNP markers is an important indicator for evaluating genetic maps. Collinearity analysis revealed that > 90% of the SNP loci on the linkage map were in the same order as those on the corresponding chromosomes of the physical map (Fig. 3), making the annotation of genes within QTL intervals feasible.

### Mapping of MFSL

Based on the high-density genetic map, nine QTLs associated with MFSL were mapped on seven LGs through inclusive composite interval mapping (ICIM), and QTLs were not detected on LG A05, 07 and 08 (Table 3). Of the nine QTLs, two were detected on LG A01 (MFSL1.1Y2016 and 1.2Y2017) and 03 (MFSL3.1Y2016 and 3.2Y2017), respectively, and one each on LG A02, 04, 06, 09 and 10. Among nine detected MFSL loci, two loci (MFSL4 and 10) were identified across two years and called stable QTLs, and the rest were environment-specific QTLs. The phenotypic variance explained by a single QTL ranged from 4.10% (MFSL9/Y2016) to 22.13% (MFSL4/Y2016), and the logarithm of the odds (LOD) score ranged from 3.11 to 14.49. The two largest phenotypic variances were LG A04 (MFSL4) and A10 (MFSL10), whereas the environment-specific QTL showed smaller phenotypic variance in QTL detected each year. The additive effects were negative for all MFSL QTL, indicating that increased trait values were conferred by the female (He102) alleles. Because the two stable QTLs, MFSL4 and MFSL10, had higher phenotypic variance, these were also recognized as major-effect QTLs. The adjacent markers for MFSL4 and MFSL10 were Marker346068/347912 and Marker985918/992274, respectively, with linkage interval sizes of 0.60 cM and 2.08 cM and physical interval sizes of 44.37 kbp and 121.91 kbp, respectively. There were 2056 and 6769 SNPs in the respective confidence intervals according to parental lines’ resequencing results, which involved 24 and 199 mutant genes, respectively (Additional file 2: Table S2).

**Table 3:** Detailed information on the QTLs associated with MFSL during 2016 and 2017 in Chinese cabbage

QTL No.	Year	Chromosome	Peak position	LOD	PVE%
MFSL1.1	2016	A01	98	4.78	6.07
MFSL1.2	2017	A01	91	4.59	7.55
MFSL2	2016	A02	3	4.39	5.47
MFSL3.1	2016	A03	4	8.16	10.89
MFSL3.2	2017	A03	55	4.22	6.96
MFSL4	2016/2017	A04	52	14.49/8.33	22.13/14.59
MFSL6	2017	A06	150	3.11	4.89
MFSL9	2016	A09	187	3.31	4.10
MFSL10	2016/2017	A10	115	9.34/7.52	12.99/12.33

## Discussion

High-density genetic mapping is an important tool in genetics and genomics research, especially for fine mapping and map-based gene cloning. Previous studies have reported high-density linkage map construction in *B. rapa* using the SLAF-Seq strategy [2]. Yu and his colleagues have constructed a sequence-based bin map with a doubled haploid (DH) population containing 100 DH lines. The final map included 1064 bins or 3688 polymorphic loci (206 previously reported molecular markers and 3482 new SLAF markers) on 10 LGs. The map was 858.98 cM in length, with an average interlocus distance of 0.81 cM. However, the population derived from two parental lines with significant difference was used for QTL mapping only in several agronomic traits rather than all agronomic traits. For example, the DH100 population constructed by Yu et al. [2] was used for mapping QTL of downy mildew resistance in Chinese cabbage. To map more QTLs of important agronomic traits, additional mapping populations are required.

For spring-type Chinese cabbage production, premature bolting has remained a major challenge. It often causes excessive elongation of the dwarf stem and results in the loss of commercial value for spring-type Chinese cabbage leaf-head. Because the elongated dwarf stem would develop into main flower stalk, we selected parental lines with significant differences in MFSL (Fig. 1) for mapping population construction. However, previous studies about QTL mapping of bolting-resistant traits seldom focused on MFSL.

To construct high-resolution genetic map, a total of 150 RILs were used in our study. To obtain high-quality SNP markers for linkage map construction, the method of combining deep resequencing on parental lines and SLAF-Seq on offspring individual line was conducted. Here, it was unnecessary to sequence the parental lines for linkage map construction and QTL mapping. However, more polymorphic markers were required for fine mapping of major-effect QTL and map-based cloning of candidate genes. In fact, our strategy resulted in 1,082,885 SNP markers between parental lines, of which 805,813 showed an aa×bb segregation pattern. These high-quality SNP markers provide a marker pool for fine mapping and map-based cloning of candidate genes in subsequent studies.

To construct a reliable linkage map, the isolated aa×bb pattern SNP markers still need stringent screening. High-depth detection in parental lines could ensure accurate genotyping of offspring individuals. In this research, the parameter setting on the depth of the selected SNP markers was not only larger than 14× in parental lines, but also larger than 5× in at least 90% (the value of integrity) of offspring lines. Actually, the average depth of the selected markers in two parental lines and offspring was 34.18× and 27.98×, respectively. It was significantly larger than previous study provided by Yu et al. [2] in which the average depth of parental lines and offsprings was only 21.96× and 10×, respectively. The integrity of all selected markers was 99.96% on average, significantly > 80% in the previous one [2]. Otherwise, to make sure that the annotation of genes within QTL intervals was feasible, collinearity analysis was performed (Fig. 3). Finally, the linkage map included 5392 high-fidelity SNP markers on 10 LGs, with total map length of 1687.82 cM and an average distance of 0.32 cM between adjacent markers (Fig. 2 and Table 2), which were significantly larger and denser than previously constructed bin map [2]. Significant differences in total map length were existed between the present study and Yu et al. [2]. It might be caused by the difference in parental lines, mapping population, population size and total marker number [23, 24]. The parental lines we selected showed great polymorphism, the RIL population could produce a large number of recombination events, the size RIL150 mapping population was larger than previous DH100, and the total number of mapping marker in present study was also significantly more than previously one [2]. To our knowledge, this map has the highest marker-density to date among individual experimental Chinese cabbage genetic maps.

Based on the present high-density linkage map, high-resolution mapping on QTLs of MFSL was performed. In a previous study, QTL mapping of bolting-related traits in *B. rapa* showed that all 10 LGs cover QTLs, except for LG A04 [17]. This suggested that a novel QTL related to bolting was identified based on present phenotypic indices, namely, the length of main flower stalk. MFSL10 was the other major-effect QTL found in the current study, it might be localized to the bottom of LG A10 according to the ratio of peak to total map length (115/128), which may be applicable to the qDB10–2 previously determined [17]. The flower stalks here were similar to the inflorescence stems in *Arabidopsis*. In previous studies involving QTL mapping of bolting-resistant traits, the indices such as BT [11, 18], FT [14, 15, 17, 19, 20], DB [8, 17], and BI [13, 17, 21] were concerning whether with flower buds or first flower opening. However, extensive investigations on *Arabidopsis* revealed that the development of flower and inflorescence stem involves different molecular mechanism [25], and both processes are influenced by the same environmental factors such as temperature and illumination.

QTL mapping for plant height (PH) in *B. rapa* was conducted using chromosome segment substitution lines (CSSLs), and a total of 14 QTLs for PH were identified, but none were located on chromosome A04 [16]. In the study, the PH was measured from the ground to the top of the stem on the day the first flower blossomed. The measuring method of MFSL was similar to PH, but the measuring time was different. Because the first bud blossomed at different times for different lines, the PH value for different lines could not be compared with each other. The notion of PH was related to the blossom of first flower, it might be closely linked with flowering time. The annual harvest time of spring-type Chinese cabbage is fixed. One of most important commercial character for harvested spring-type Chinese cabbage was whether the dwarf stem was overly elongated or not. The excessive elongation of dwarf stem would cause a decrease in the product value. In practice, mature spring-type Chinese cabbage leaf-heads with shorter dwarf stems were of higher demand than those with longer dwarf stems. Therefore, it is necessary to identify markers closely linked with shorter dwarf stems, which will develop into the main flower stalk, for breeding bolting-resistant spring-type Chinese cabbage by MAS. The present work focused on achieving this goal. Moreover the confidence intervals of two stable QTLs identified in present study were away from distance of SDM, it made the results more credible.

The regulatory mechanism of flower stalk elongation is relatively complex. To understand the molecular mechanism underlying flower stalk elongation, candidate genes should be isolated in the future. Candidate genes were screened between the confidence intervals of major-effect QTLs, namely MFSL4 and MFSL10. SNP markers and mutant genes between the confidence intervals were identified. In our future study, we plan to conduct fine-mapping and map-based candidate gene identification.

## Conclusions

In this study, a high-density genetic map of *B. rapa* was constructed using the SLAF-Seq technique. To our knowledge, this map has the highest marker density in *B. rapa* to date. Furthermore, nine QTLs associated with MFSL, including two major-effect QTLs, were identified. Notably, MFSL04 is a novel QTL associated with bolting mapped on LG A04 based on the MFSL. SNP markers and mutant genes between the confidence intervals were also analyzed. These results lay a foundation for fine-mapping and map-based candidate genes identification for flower stalk length.

## Methods

### Mapping population and phenotyping

A RIL population consisted of one hundred and fifty F_2:7_ recombination inbred lines derived by single-seed descent from a cross between two parents, 06–247 and He102, was used. 06-247, the maternal parent with shorter dwarf stems, was selected from the selfing progenies of Japanese hybrid JianChun (JingShenCai No. 2004007, imported from TAKII Seed Company, Japan). He102, the paternal line with longer dwarf stems, is a selfing progeny of a local variety, Henan Erbaotou, collected from Henan Province in China [26].

Phenotypic experiments were conducted at the Core Experiment Station of the Vegetable and Flower Institute of Shandong Academy of Agricultural Sciences, Jinan, China (36.67 N, 116.98 E) during two spring growing seasons from 2016 to 2017. The seeds of each line were germinated at 28 °C for 24–48 h in darkness, and the germinated seeds were sown into 7-cm pots on January 3, 2016. Five plants were selected as one independent biological replicate, and three biological replicates were used for randomized complete block design. The pots were directly placed in a solar greenhouse. In 2016, the greenhouse was covered by a layer of thermal insulating layer when the natural temperature descended below 0 °C, at night, whereas in 2017, the greenhouse was covered by two layers of plastic film that were separated by a 50-cm interval to keep warm. Generally, the condition in the greenhouse was 6–8 h day (highest to 20 °C) /16–18 h night (lowest to 3 °C) with a light intensity of 8000–16,000 lx during both growing seasons before transplanting. On March 10th of each year, all plantlets were transplanted in a pest-prevent net (φ0.8) room until seeds were harvested. The main flower stalk was measured from the ground to the top of the stem after 30 days of transplanting at each growing season. The mean phenotypic data, standard deviation and correlation analysis were performed by microsoft excel. The mean phenotypic data for three replicates of the individual was used for further analysis. The lines with phenotypic data less than three replications were deleted and a final 136 lines were used for QTL mapping.

### DNA extraction, library preparation, and sequencing

For DNA extraction, the leaves of the parents and the RIL population were sampled and frozen in liquid nitrogen. High-quality genomic DNA was extracted using the DNeasy Plant MaxiKit (Qiagen, USA) and stored at − 20 °C prior to preparation of a sequencing library.

Two different sequencing library construction strategies were used for parent lines and offsprings. For parent lines, standard paired-end (PE) libraries with 350-bp inserts were constructed following the manufacturer’s instructions (Illumina, USA). Briefly, extracted gDNA was sheared ultrasonically using a Covaris system (Applied Biosystems, USA). Then, the fragmented DNA was purified with the QIAquick PCR Purification Kit (Qiagen, USA), followed by end-repair and index adapter ligation using the TruSeq PE Cluster Kit v3-cBot-HS (Illumina, USA). The resulting libraries were size-selected on a 2% agarose gel. The fragments between 300 bp and 500 bp were collected with the QIAquick Gel Extraction Kit (Qiagen, Germany) and used for amplification by polymerase chain reaction (PCR). The resulting PCR products were purified further and used for the sequencing library. For the RIL population, the SLAF-Seq strategy with minor modifications was used according to Sun et al. [22], in which two enzymes (*Rsa*I and *Hae*III, New England Biolabs, NEB, USA) were used to digest the genomic DNA. The details of the following operation were referred to in Yu et al. [2]. All high-throughput sequencing was performed on an Illumina HiSeq 2500 system (Illumina, USA) according to the manufacturer’s recommendations. The sequencing and the following bioinformatics analysis were conducted by Biomarker Technologies (Beijing, China).

### Sequence data analysis

The sequence data of the parental lines and each RIL were distinguished by Illumina Casava 1.8 in FASTQ format. The raw reads were first sorted according to barcodes. Then a series of quality control (QC) procedures were adopted. QC standards were performed using the Trimmomatic program as follows [27]: (1) Reads with > 10 nt aligned to the adapter were removed, (2) Reads containing the *Rsa*I or *Hae*III sequences were removed, (3) Reads with unidentified nucleotides (Ns) > 10% were removed, (4) Reads with > 50% of bases having a *Q*_phred_ < 20 were removed (*Q*_phred_ can be used to represent average error rate (e), the formula between *Q*_phred_ and e is as follows: *Q*_phred_ = − 10*log_10_*e*.). The sequencing depth for each base and total reads coverage compared to the reference genome were calculated based on the alignments.

### Mapping reads and SNP calling

Sequence reads were aligned to the *B. rapa* reference genome V1.5 (http://brassicadb.org/brad/) [28] with the Burrows-Wheeler Aligner (BWA) using default parameters. Briefly, unmapped reads or the reads mapped to multiple locations were excluded. The aligned reads considered to be PCR duplicates were removed using the MarkDuplicates in the Picard software package. At last, a series of processes were performed using the Genome Analysis Toolkit (GATK) to identify polymorphic sites: (1) Regions near short insertion or deletion (InDel) were realigned locally with IndelRealigner; (2) Base quality scores were recalibrated with Base Recalibration; (3) Variants such as SNPs and InDels were extracted with UnifiedGenotyper; (4) SNP clusters (two SNP in between a 5-bp region) and SNPs within 5-bp regions adjacent to In/Dels were removed. The resulting SNP markers were used for the following genotyping and mapping analysis.

### SNP marker selection and genotyping of RIL population

To obtain high-quality SNP markers for genetic map construction, all SNPs identified among parental lines and the offspring were filtered as follows: markers with three or more kinds of base type, markers showing no polymorphisms between parental lines, markers not detected or with a depth of < 4× in the parental lines. The remaining polymorphic markers were potential markers suitable for subsequent analysis and they were classified into eight segregation patterns (ab×cd, ef × eg, hk × hk, lm × ll, nn × np, aa×bb, ab×cc, and cc × ab). The mapping population was a RIL population, so tags showing the aa×bb segregation pattern were used for genetic map construction. For high-confidence genetic map construction, the resulting markers with integrity (defined as the percentage of determined markers for all mapped markers in a given line) < 90% and sequencing depth less than 14× in the parental lines as well as < 5× in the offspring were further filtered.

### High-density genetic map construction and segregation distortion markers

Based on the genotyping data of 150 RILs, a high-density genetic map comprising 10 LGs was constructed by a newly developed HighMap strategy [29]. The detailed protocol of HighMap contains four modules, designed for linkage grouping, marker ordering, error genotyping correction and map evaluation, respectively. In order to produce a high-confidence genetic map, the ordering module and error genotyping correction module were conducted iteratively. Map distances were estimated using the Kosambi mapping function [30]. Segregation distortion markers were unavoidable. Marker segregation ratios were calculated using the chi-square test. The markers showing significant (*p* < 0.05) segregation distortion were initially eliminated when the linkage map was constructed. Error genotypes of segregation distortion markers were corrected further by the SMOOTH software [31] according to parental contribution of genotypes, and then missing genotypes were imputed based on a k-nearest neighbor algorithm [32]. They were then added as accessory markers.

### Collinearity analysis between the genetic and physical maps

All the sequences of SNP markers that were constructed in the linkage map were aligned back to the physical sequence of the *B. rapa* genome through local Basic Local Alignment Search Tool (BLAST) to confirm their physical positions in the genome. Software CIRCOS 0.66 was used to compare the collinearity of markers based on their genetic positions and physical positions [29].

### MFSL evaluation and QTL mapping analysis

The MFSLs of the two parents were compared by the Student’s *t*-test at the 5 and 1% levels of probability. The frequency distribution of MFSL in RIL population was analyzed. Inclusive composite interval mapping in the bi-parental populations (BIP) model of QTL IciMapping software v4.0 [33] was used to detect the additive QTLs for MFSL with the *P* values for entering variables (PIN) = 0.001. Because many adjacent markers in the SNP linkage map had a map distance of < 1 cM, the scanning step was 1.0 cM. LOD significance thresholds for QTL peaks were determined using 1000 permutations. The percentage of variance explained and the additive effect for each QTL were also estimated.

## Additional files

Additional file 1: **Table S1.** Phenotypic analysis of MFSL in two parents and RIL population of Chinese cabbage. (XLSX 95 kb)

Additional file 2: **Table S2.** SNP markers and mutant genes between the confidence intervals identified in Chinese cabbage. (XLSX 736 kb)

## Data Availability

The data that support the findings of this study are available from the repository of NCBI Sequence Read Archive (SRA) with the accession numbers: SRR9095322, SRR9095323, SRR9095324, SRR9095325, SRR9095326, SRR9095327, SRR9095328, SRR9095329, SRR9095330, SRR9095331, SRR9095332, SRR9095333, SRR9095334, SRR9095335, SRR9095336, SRR9095337, SRR9095338, SRR9095339, SRR9095340, SRR9095341, SRR9095342, SRR9095343, SRR9095344, SRR9095345, SRR9095346, SRR9095347, SRR9095348, SRR9095349, SRR9095350, SRR9095351, SRR9095352, SRR9095353, SRR9095354, SRR9095355, SRR9095356, SRR9095357, SRR9095358, SRR9095359, SRR9095360, SRR9095361, SRR9095362, SRR9095363, SRR9095364, SRR9095365, SRR9095366, SRR9095367, SRR9095368, SRR9095369, SRR9095370, SRR9095371, SRR9095372, SRR9095373, SRR9095374, SRR9095375, SRR9095376, SRR9095377, SRR9095378, SRR9095379, SRR9095380, SRR9095381, SRR9095382, SRR9095383, SRR9095384, SRR9095385, SRR9095386, SRR9095387, SRR9095388, SRR9095389, SRR9095390, SRR9095391, SRR9095392, SRR9095393, SRR9095394, SRR9095395, SRR9095396, SRR9095397, SRR9095398, SRR9095399, SRR9095400, SRR9095401, SRR9095402, SRR9095403, SRR9095404, SRR9095405, SRR9095406, SRR9095407, SRR9095408, SRR9095409, SRR9095410, SRR9095411, SRR9095412, SRR9095413, SRR9095414, SRR9095415, SRR9095416, SRR9095417, SRR9095418, SRR9095419, SRR9095420, SRR9095421, SRR9095422, SRR9095423, SRR9095424, SRR9095425, SRR9095426, SRR9095427, SRR9095428, SRR9095429, SRR9095430, SRR9095431, SRR9095432, SRR9095433, SRR9095434, SRR9095435, SRR9095436, SRR9095437, SRR9095438, SRR9095439, SRR9095440, SRR9095441, SRR9095442, SRR9095443, SRR9095444, SRR9095445, SRR9095446, SRR9095447, SRR9095448, SRR9095449, SRR9095450, SRR9095451, SRR9095452, SRR9095453, SRR9095454, SRR9095455, SRR9095456, SRR9095457, SRR9095458, SRR9095459, SRR9095460, SRR9095461, SRR9095462, SRR9095463, SRR9095464, SRR9095465, SRR9095466, SRR9095467, SRR9095468, SRR9095469, SRR9095470, SRR9095471, SRR9095472, SRR9095473 (http://www.ncbi.nlm.nih.gov/bioproject/542546).

## Abbreviations

BI: Bolting index
BT: Bolting time
DB: Days to bolting
DTF: Days-to-flowering
FT: Flowering time
ICIM: Inclusive composite interval mapping
LGs: Linkage groups
MAS: Marker-assisted selection
MFSL: Main flower stalk length
QTL: Quantitative trait loci
RIL: Recombinant inbred line
SLAF-Seq: Specific-locus amplified fragment sequencing
SNPs: Single nucleotide polymorphisms
